# Risk factors of atrial fibrillation complicated with cognitive impairment and the relationship between cardiac function parameters and the degree of cognitive impairment

**DOI:** 10.1016/j.clinsp.2024.100453

**Published:** 2024-08-28

**Authors:** FengJiao Liao, ZongYi Hou

**Affiliations:** Department of Neurology, The First People's Hospital of Pinghu City, Pinghu City, Zhejiang Province, China

**Keywords:** Atrial fibrillation, Cognitive dysfunction, Risk factors, Cardiac function, Correlation analysis

## Abstract

**Objective:**

To explore the risk factors of Atrial Fibrillation (AF) with Cognitive Impairment (CI) and to analyze the relationship between cardiac function parameters and the degree of CI in patients.

**Methods:**

120 AF patients were selected, and Montreal Cognitive Assessment (MoCA) was used to distinguish between AF patients with and without CI. Univariate analysis and multivariate Logistic regression analysis were used to evaluate the impact of sociodemographic data, disease-related data, and clinical data on risk factors for AF with CI. Pearson's method was used to analyze the correlation between cardiac function parameters and cognitive function scores in AF patients.

**Results:**

There were 89 patients with CI and 31 patients without CI, and the MoCA scores of patients with CI were lower than those in patients without CI. Age, occupational status, educational level, combined smoking history, drinking history, and heart failure, as well as systolic blood pressure, diastolic blood pressure, total cholesterol, triglyceride, C-reactive protein, free thyroxine, free triiodothyronine, and D-dimer were risk factors for the patient with CI. Left atrial diameter, left ventricular end-diastolic diameter, left ventricular end-systolic diameter, and left atrial maximum volume in patients with CI were higher than those in patients without CI, and left ventricular ejection fraction and peak early diastolic velocity/peak late-diastolic mitral velocity ratio were lower.

**Conclusion:**

The cardiac function parameters of patients are closely related to attention, orientation, memory, visuospatial, and executive ability. Cardiac function parameters were closely related to cognitive functions.

## Highlights

• Pathological characteristics of atrial fibrillation with cognitive impairment.
• Correlation between cardiac function and degree of cognitive impairment.
• Risk factors for cognitive impairment associated with atrial fibrillation.

## Introduction

Atrial Fibrillation (AF) is a tachyarrhythmia that is characterized by rapid and disorderly fibrillation waves replacing regular and orderly electrical activity, which often leads to cardiac insufficiency, sudden cardiac death, cerebral artery embolism, and all-cause death.^1^^,^^2^ Epidemiological surveys show that the prevalence of AF is about 0.1 % in adults under 60 years old, 5.8 % in adults 70‒79 years old, and as high as 9 % in people over 80 years old.^3^^,^^4^ With the increasing prevalence and incidence of AF in recent years, serious comorbidities and mortality have gradually increased, resulting in a huge health and economic burden on families and society. Randomized Controlled Trials (RCTs), prospective studies, and meta-analyses have shown that AF is closely related to Cognitive Impairment (CI) disease progression and can develop into dementia.^5^ AF patients have a 1.4-fold increased risk of CI and a 1.3-fold increased risk of dementia. AF is a strong independent risk predictor of patients with CI, which increases the hospitalization rate, disability rate, and mortality rate.^6^^,^^7^ CI includes Mild CI (MCI) and dementia, and is an important disease that seriously endangers the health of middle-aged and elderly people.^8^ About 40 % of AF patients are diagnosed with MCI or dementia, and the risk is 2.25 and 1.28 times higher, respectively than in the normal population.^9^ At present, there is no effective cure for CI, and the resulting loss of orientation, disability, loss of exercise ability, and reduced ability of daily living seriously affect the health and quality of life of patients.^10^ Therefore, exploring the risk factors for CI in AF patients is of great significance for early prevention or delay of the occurrence and progression of CI.^11^ In recent years, some scholars have discussed the risk factors of patients with CI. Domestic and foreign research reports have shown that incidence type, hypertension level, diabetes, hemoglobin levels, age, gender, education level, cerebrovascular history, heart failure, left atrial size, etc. may be risk factors for CI in AF patients. In addition, Echocardiography has been widely used to assess changes in local and global cardiac function.^12^ The method is low-cost, non-invasive, and real-time. In particular, current high-frequency ultrasound has a higher temporal resolution. By adjusting the scanning angle and sampling depth, the motion information of different myocardium segments during the whole cardiac cycle can be obtained, so as to evaluate the cardiac condition more comprehensively.^13^ The purpose of this study was to investigate the risk factors of AF with CI and to analyze the relationship between cardiac function parameters and the degree of CI in patients, so as to provide an effective basis for clinical early prevention or delay of CI in AF patients.

## Materials and methods

### Research objects

AF patients diagnosed and treated at The First People's Hospital of Pinghu City from January 2020 to May 2022 were selected as research subjects, with a total of 120 subjects. Reporting follows the STROBE guidelines for reporting on observational studies. The standard 12-lead electrocardiogram was employed for the diagnosis of AF. Clinical diagnosis of AF can be made when the standard 12-lead electrocardiogram or a single lead electrocardiogram (lasting ≥30s) reveals the absence of a normal P-wave, which is instead replaced by a sequence of f waves exhibiting varying shapes, sizes, and intervals, while maintaining normal atrioventricular conduction function as indicated by RR intervals.^14^^,^^15^

Inclusion criteria: (1) All subjects underwent a 24-hour Holter ECG recording using the ambulatory electrocardiographic recorder SpiderView (Sorin Group, Italy). The diagnosis of AF was made in accordance with the definitions of the 2016 European Society of Cardiology guidelines; (2) Patients ≥18 years old; (3) All subjects or their family members were aware of the research content and signed the informed consent. Exclusion criteria: (1) Patients with AF caused by trauma and surgery; (2) patients with other cardiac organic diseases; (3) Patients with autoimmune diseases; (4) Patients with malignant tumors; (5) Patients with severe liver and kidney insufficiency; (6) Patients with hematological diseases; (7) Patients with abnormal thyroid function; (8) Patients with mental disorders or unable to complete the cognitive test. All contents and methods of this study were approved by the ethics committee of The First People's Hospital of Pinghu City (n° 201805ZJ5001). The necessity of anticoagulant therapy and the duration of treatment should be determined based on the CHA2DS2 Vasc score. A CHA2DS2 Vasc score of 2 or higher indicates a requirement for anticoagulant therapy, with the option to choose between warfarin or new oral anticoagulants. A score of 1 may warrant aspirin antiplatelet aggregation therapy or no anticoagulant treatment, while a score of 0 indicates that anticoagulant therapy is not necessary.

### Cognitive function assessment

Montreal Cognitive Assessment (MoCA) was used to evaluate the cognitive function of AF patients, including orientation ability (6-points), visuospatial and executive ability (5-points), naming ability (3-points), memory ability (5-points), attention ability (6 points), language ability (3-points), abstraction ability (2-points). The full score of MoCA is 30 points, and the higher the score, the better the cognitive ability. Patients with MoCA score ≥26 were considered to have normal cognitive function^16^ (AF without CI), otherwise they were considered to have cognitive dysfunction (AF with CI).

### Risk factors

Data of all subjects were collected. (1) Socio-demographic data: gender, age, marital status, occupational status, and educational level; (2) Disease-related data: smoking history, drinking history, disease type, disease course, combined with hypertension, coronary heart disease, diabetes, chronic obstructive pulmonary disease, and heart failure; (3) Relevant clinical data: body mass index, blood pressure (systolic and diastolic), blood routine indicators (hemoglobin, platelets, red blood cells, and white blood cells), blood lipid levels (total cholesterol, triglycerides, high-density lipoprotein, and low-density lipoprotein), C-reaction protein, thyroid-related hormones (free thyroxine, free triiodothyronine, and thyroid-stimulating hormone), D-dimer, uric acid, blood urea nitrogen, and creatinine.

### Detection of cardiac function parameters

The cardiac function parameters of all subjects were detected using a DW-CE 540 color Doppler ultrasound system. The long axis of the left ventricle near the sternum was taken, and the vertical line from the distal posterior wall of the aorta to the intima of the posterior wall of the left atrium was taken. Cardiac structure parameters include left atrial diameter, left ventricular end-diastolic diameter, left ventricular end-systolic diameter and left atrial volume index. Cardiac function parameters include peak early diastolic velocity and peak late-diastolic mitral velocity. Apical four-chamber and two-chamber views were taken, and left ventricular ejection fraction was calculated using the Simpson method. All parameters were continuously evaluated for 5‒7 cardiac cycles and averaged.

### Statistical analysis

All data statistics and analyses were performed using SPSS 26.0 software. Measurement data were expressed as mean ± Standard Deviation (SD), and *t*-test was used for comparison between two groups, and univariate analysis was used for comparison between multiple groups. Enumeration data were expressed as [n], and data analysis was performed using the chi-square test. The occurrence of cognitive dysfunction (a MOCA score < 26) in AF patients was used as the dependent variable (the absence of cognitive dysfunction was assigned a value of 1, and the occurrence of cognitive dysfunction was assigned a value of 0), and the remaining potential parameters were used as independent variables. One-way ANOVA was used to screen independent variables that had an impact on the occurrence of CI, and the statistically significant independent variables (risk factors) were included in a multivariate logistic regression model to describe the OR value and 95 % CI. Pearson method was used for correlation analysis; p < 0.05 was considered statistically significant.

## Results

### General information

Of the 120 AF patients included in this study, there were 54 males and 66 females, aged (61.78 ± 9.32) years. Compared with patients with AF without CI, patients with AF with CI were characterized by higher age, employment, and lower education level (*p* < 0.001, *p* < 0.05, *p* < 0.01). In addition, there was a statistical difference (*p* < 0.05) between AF patients with and without CI in terms of BMI, smoking history, alcohol consumption history, and comorbid heart failure (Table 1). There were no statistically significant differences between the two groups in terms of gender, marital status, type/duration of AF, medical history (including hypertension, coronary heart disease, and chronic obstructive pulmonary disease), and CHA2DS2-Vasc score (p > 0.05).

**Table 1:** Univariate analysis of sociodemographic data of patients with atrial fibrillation complicated by cognitive impairment.

	AF with CI (*n* = 89)	AF without CI (*n* = 31)	*χ*^2^ value	p-value
Gender (n)			0.19	0.6598
Male	39	15		
Female	50	16		
Age (mean ± SD, year)	63.46 ± 8.93	56.94 ± 8.84		0.0006***
BMI (kg/m^2^)	23.16 ± 1.77	23.36 ± 1.83		0.6031
Marital Status (n)			1.67	0.6432
Unmarried	12	6		
Married	68	23		
Divorced	7	1		
Widowed	2	1		
Occupational Situation (n)			7.32	0.0257*
On-the-job	54	27		
Retire	27	3		
Other	8	1		
Education Level (n)			15.37	0.0015**
Elementary School and Below	8	0		
Junior High School	22	1		
High School	28	8		
College and Above	31	22		
Smoking History (n)			7.97	0.0186*
No	37	22		
Yes	33	6		
Quit	19	3		
Drinking History (n)			6.92	0.0313*
No	32	18		
Yes	44	7		
Quit	13	6		
AF Type (n)			2.79	0.2478
Paroxysmal	25	11		
Persistent	36	13		
Permanent	28	7		
AF Course (n)			4.04	0.2578
< 1 year	25	8		
1‒5 year	17	15		
6‒10 year	18	8		
> 10 year	29	0		
High Blood Pressure (n)			2.10	0.1473
No	44	20		
Yes	45	11		
Coronary Heart Disease (n)			2.25	0.1339
No	62	17		
Yes	27	14		
Diabetes (n)			3.27	0.0704
No	54	13		
Yes	35	18		
Chronic Obstructive Pulmonary Disease (n)			0.28	0.5944
No	48	15		
Yes	41	16		
Heart Failure (n)			4.02	0.0449*
No	36	19		
Yes	53	12		
CHA2DS2-Vasc score			0.891	0.641
0	36	12		
1	15	5		
≥ 2	28	14		

Significance was considered at values of *p* < 0.05.

### Evaluation of AF with CI in patients

To evaluate whether patients with AF have CI, MoCA was performed on all subjects. The MoCA scale assessed a number of cognitive domains in the study population. As shown in Table 2, the MoCA scores were (19.68 ± 2.83) in patients with CI and (27.58 ± 1.41) in patients without CI. The MoCA scores of patients with CI were significantly lower than those of patients without CI (*p* < 0.0001). In addition, patients with AF with CI also had lower scores in all cognitive domains than patients with AF without CI (Table 2).

**Table 2:** MoCA score of atrial fibrillations with cognitive impairment in patients.

	AF with CI	AF without CI	p-value
MoCA total score	19.68 ± 2.83	27.58 ± 1.41	<0.0001***
Orientation	3.44 ± 0.81	5.32 ± 0.46	<0.0001***
Visuospatial and executive	2.89 ± 0.87	4.71 ± 0.45	<0.0001***
Object naming	2.13 ± 0.84	2.81 ± 0.39	<0.0001***
Memory	3.15 ± 0.84	4.58 ± 0.49	<0.0001***
Attention	3.80 ± 1.28	5.29 ± 0.45	<0.0001***
Language	2.51 ± 0.72	2.84 ± 0.37	0.0161*
Abtraction	1.79 ± 0.41	1.97 ± 0.18	0.0196*

Significance was considered at values of *p* < 0.05.

### Univariate analysis of data related to AF with CI in patients

Next, blood pressure levels and laboratory measures were compared between the two groups. The results showed that systolic blood pressure (*p* < 0.01), diastolic blood pressure (*p* < 0.05), total cholesterol (*p* < 0.01), triglyceride (p < 0.05), C-reactive protein (*p* < 0.05), free thyroxine (*p* < 0.01), free triiodothyronine (*p* < 0.05), and D-dimer (*p* < 0.001) were significantly higher in patient with CI than those in patients without CI (Table 3).

**Table 3:** Blood pressure level and laboratory measurement indicators of atrial fibrillation patients with cognitive impairment and those without cognitive impairment.

	AF with CI	AF without CI	p-value
SBP	130.99 ± 7.32	126.25 ± 7.15	0.0025**
DBP	84.71 ± 9.36	79.83 ± 8.25	0.0122*
HGB	134.71 ± 7.50	79.83 ± 8.26	0.0674
PLT	211.94 ± 47.28	216.61 ± 39.14	0.6251
RBC	4.48 ± 0.26	4.42 ± 0.32	0.3730
WBC	7.41 ± 1.04	7.36 ± 1.03	0.7968
TC	4.63 ± 0.59	4.29 ± 0.53	0.0049**
TG	1.25 ± 0.31	1.12 ± 0.21	0.0327*
HDL-C	1.31 ± 0.18	1.30 ± 0.12	0.7084
LDL-C	2.55 ± 0.22	2.46 ± 0.25	0.0838
FT4	8.43 ± 1.49	7.78 ± 1.02	0.0013**
FT3	18.19 ± 2.76	16.43 ± 1.72	0.0267*
TSH	5.33 ± 0.74	4.99 ± 0.65	0.7928
D-D	3.44 ± 0.60	3.40 ± 0.56	<0.0001***
UA	249.27 ± 52.35	25325 ± 42.86	0.7078
BUN	5.01 ± 1.25	4.63 ± 1.20	0.1414
Cr	78.94 ± 9.47	79.93 ± 9.57	0.6207

SBP, Blood Pressure including Systolic Blood Pressure; DBP, Diastolic Blood Pressure; HGB, Blood Routine including Hemoglobin; PLT, Platelets; RBC, Red Blood Cells; WBC, White blood cells; TC, Total cholesterol; TG, Triglyceride; HDL-C, High density lipoprotein; LDL-C, Low density lipoprotein; CRP, C-reaction protein; FT4, Free thyroxine; FT3, Free triiodothyronine; TSH, Thyroid stimulating hormone; D-D, D-dimer; UA, Uric acid; BUN, Urea nitrogen; Cr, Creatinine. Significance was considered at values of *p* < 0.05.

### Univariate analysis of cardiac function parameters in patient with CI

**Fig. 1:**
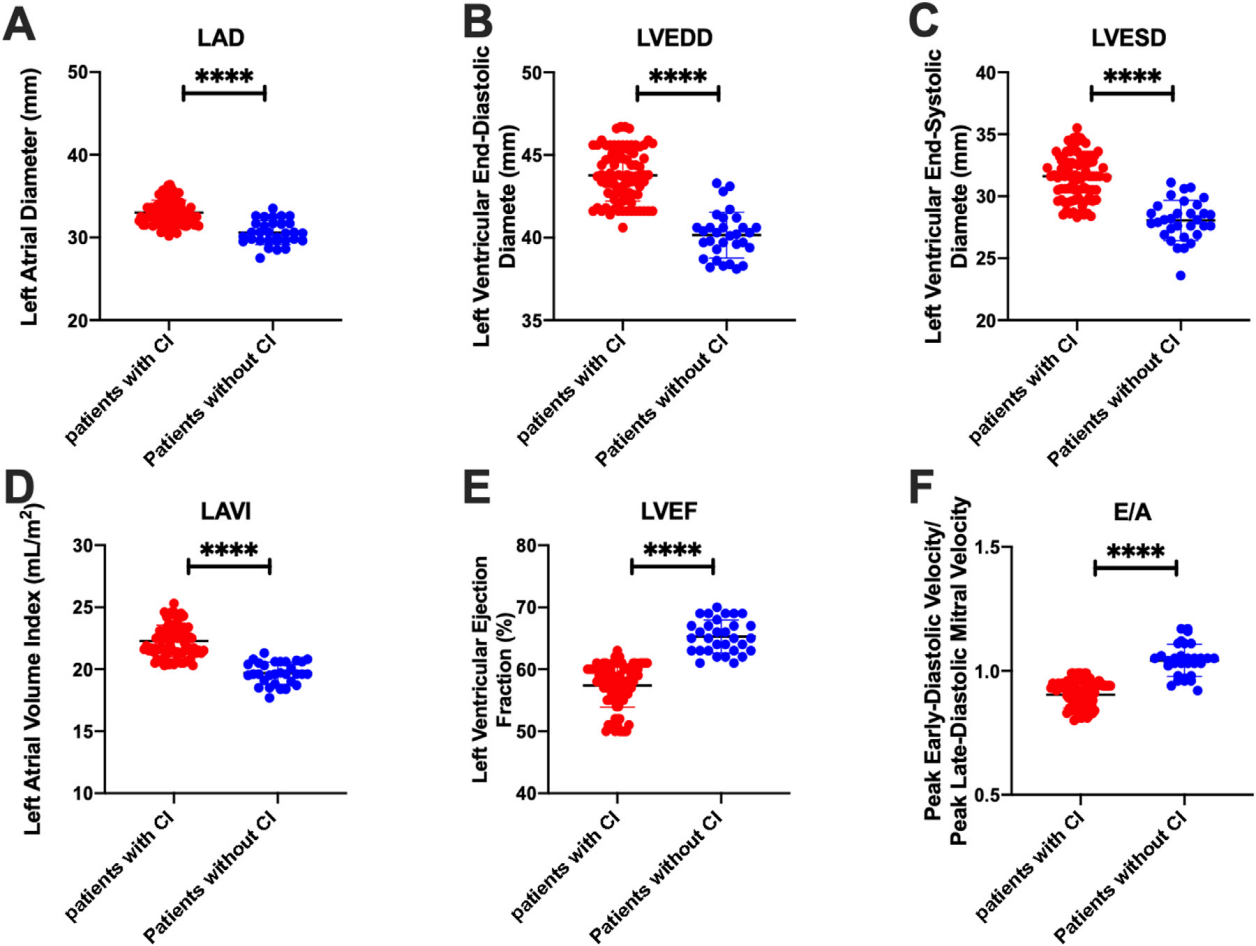
Cardiac function parameters of atrial fibrillation patients with cognitive impairment and those without cognitive impairment. (A) Left Atrial Diameter (LAD); (B) Left Ventricular End-Diastolic Diameter (LVEDD); (C) Left Ventricular End-Systolic Diameter (LVESD); (D) Left Atrial maximum Volume Index (LAVI); (E) Left Ventricular Ejection Fraction (LVEF); (F) Peak early diastolic velocity/Peak late-diastolic mitral velocity (E/A). Significance was considered at values of *p* < 0.05 (*p < 0.05; **p < 0.01; ***p < 0.001; ****p < 0.0001).

To evaluate cardiac function in patients with CI, the authors examined cardiac function parameters in all subjects. The results showed that the cardiac structure parameters in patients with CI were significantly higher than those in patients without CI, including left atrial diameter, left ventricular end-diastolic diameter, and left ventricular end-systolic diameter (*p* < 0.0001) (Fig. 1A‒C). In addition, the left atrial maximum volume index in patients with CI was significantly higher than that in patients without CI (*p* < 0.0001) (Fig. 1D), and the left ventricular ejection fraction was significantly lower (*p* < 0.0001) (Fig. 1E). The authors assessed the ratio of early diastolic peak velocity to late diastolic peak mitral valve velocity in patients over multiple cardiac cycles. Findings indicated that individuals with CI (*n* = 31) exhibited significantly lower early diastolic peak velocity/late diastolic peak mitral valve velocity compared to those without CI (*n* = 83) (*p* < 0.0001) (Fig. 1F).

### Multivariate Logisitic regression analysis of data related to AF with CI patients

Multivariate Logisitic regression analysis of related risk factors in patient with CI was further performed. In order to exclude the influence of confounding variables on AF patients complicated with CI, multivariate Logistic regression analysis was performed on the indicators with differences in univariate analysis. The results showed junior age, occupational situation, higher education level, and lower total cholesterol and D-dimer levels were both protective against CI (*p* < 0.05, *p* < 0.001) (Table 4).

**Table 4:** Multivariate Logisitic regression analysis of disease-related data of patients with atrial fibrillation complicated by cognitive impairment.

Independent Variable	Regression Coefficients	Standard Error	*Wald χ*^2^ value	p-value	OR value (95 % CI)
Age	-0.09	0.04	5.80	0.016*	0.91 (0.85‒0.98)
Occupational Situation					
On-the-job	-20.88	0.96	471.11	<0.001***	‒
Retire	-21.20	0.00	‒	‒	‒
Other	‒	‒	‒	‒	‒
Education Level					
Elementary School and Below	-42.75	0.00	‒	<0.001***	‒
Junior High School	-21.44	1.71	158.10	<0.001***	‒
High School	-1.27	0.64	3.92	0.048*	0.28 (0.08‒0.99)
College and Above	‒	‒	‒	‒	‒
Smoking History					
No	1.23	0.78	2.52	0.112	3.42 (0.75‒15.62)
Yes	1.68	1.08	2.40	0.121	5.37 (0.64‒44.85)
Quit	‒	‒	‒	‒	‒
Drinking History					
No	-0.22	0.66	0.12	0.734	0.80 (0.22‒2.91)
Yes	-0.57	0.87	0.43	0.514	0.57 (0.10‒3.14)
Quit	‒	‒	‒	‒	‒
Heart Failure					
No	-0.76	0.65	1.37	0.241	0.47 (0.13‒1.67)
Yes	‒	‒	‒	‒	‒
SBP	-0.11	0.06	3.77	0.05	0.90 (0.81‒1.00)
DBP	-0.06	0.04	2.23	0.14	0.94 (0.81‒1.02)
TC	-1.38	0.62	4.89	0.03*	0.25 (0.07‒0.86)
TG	0.89	1.46	0.37	0.54	2.45 (0.14‒42.90)
CRP	-0.26	0.34	0.56	0.45	0.77 (0.39‒1.52)
FT4	-0.27	0.19	1.96	0.16	0.76 (0.52‒1.11)
FT3	-0.57	0.55	1.08	0.30	0.57 (0.19‒1.66)
D-D	-23.80	5.91	16.22	<0.001***	‒

Significance was considered at values of p < 0.05.

### Correlation analysis between cardiac function parameters and cognitive dysfunction

**Figure 2:**
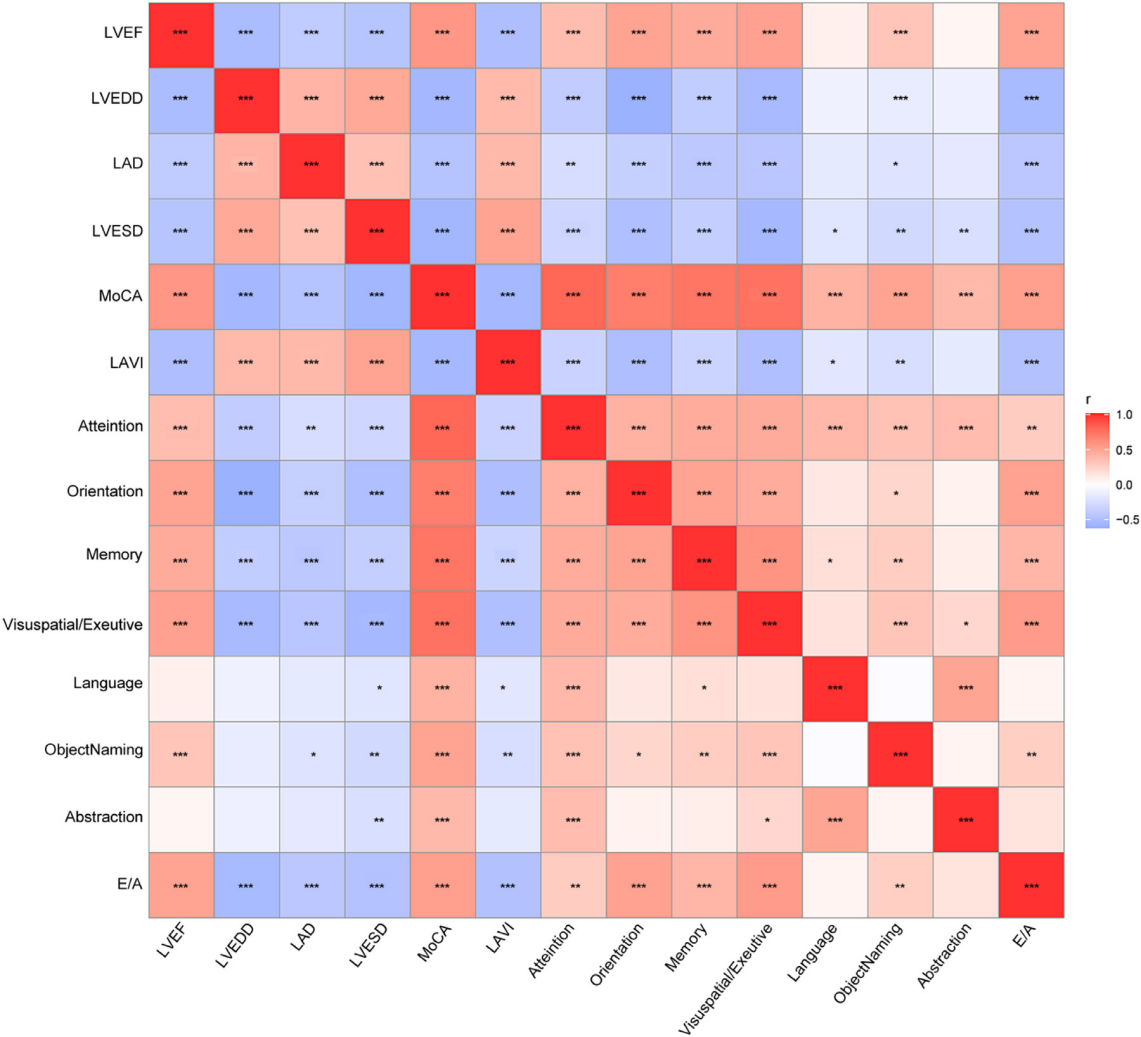
Correlation analysis of cardiac function parameters and cognitive dysfunction scores in patients with atrial fibrillation combined with cognitive dysfunction. Spearman's rank correlation was used for bivariate correlation analysis. The significance level was Bonferroni corrected at *p* < 0.05. (*p < 0.05; **p < 0.01; ***p < 0.001).

Further correlation analysis was performed on cardiac function parameters and cognitive dysfunction scores. The results showed that all cardiac function parameters were significantly correlated with MoCA scores. LAD, LVEDD, LVESD and LAVI were significantly negatively correlated with MoCA scores, whereas LVEF and E/A were significantly positively correlated with MoCA scores (*p* < 0.001) (Fig. 2). Of interest, LAD, LVEDD, LVESD, and LAVI were all significantly negatively correlated (*p* < 0.001) with attention, orientation, memory, visuospatial, and executive ability (Fig. 2).

## Discussion

AF is a common clinical arrhythmia, which can lead to atrial muscle remodeling and systolic function damage, accompanied by a variety of complications, such as heart failure, arterial embolism, ischemic stroke, etc.^17^ Cognitive dysfunction and dementia are public health issues that have attracted much attention. Patients with CI will experience functional impairment in judging objective things, thus affecting their quality of life.^18^ A retrospective cohort study based on a community population showed that cognitive function in AF patients declined more rapidly each year.^1^ This study employed the MoCA scale to evaluate 7 cognitive domains of AF patients. The present research showed that cardiac function parameters exhibited a significant correlation with cognitive functions such as attention, orientation, memory, visuospatial, and executive abilities. Additionally, several factors, such as younger age, occupational situation, higher level of education, lower total cholesterol, and lower D-dimer levels were associated with a reduced risk of CI.

Numerous studies have demonstrated a significant association between individuals diagnosed with AF and cognitive function decline.^19^, ^20^, ^21^ Furthermore, additional research has consistently indicated that the presence of AF substantially heightens the susceptibility to developing Alzheimer's disease. In a subsequent investigation conducted by Alvadori Emilia et al., it was observed that among a cohort of 128 elderly individuals with AF and CI, approximately 27 % of the patients exhibited a further decline in cognitive function.^22^ The researchers posit that this deterioration was associated with advanced age, lower educational attainment, reduced cognitive efficacy, a history of stroke, and the presence of cardiovascular disease. Studies have shown a correlation between blood indicators and cognitive function. Sun et al. found a nonlinear relationship between platelets and cognitive function scores in patients with AF.^23^ In this study, it was observed that there were no significant disparities in blood routine parameters between patients with AF accompanied by CI and those with AF without CI. It is worth noting that CI is a prevalent occurrence in diverse neurodegenerative disorders, such as Alzheimer's disease.^24^. According to a meta-analysis report, AF is a significant independent risk factor for Alzheimer's disease.^25^ In a study conducted on patients with Alzheimer's disease, the research team discovered a correlation between elevated C-reaction protein levels and impaired cognitive function.^26^ Furthermore, epidemiological studies have indicated a connection between Alzheimer's disease, inflammation, and dyslipidemia, although the precise nature of this relationship remains uncertain.^27^ This research also revealed that patients diagnosed with AF and CI exhibited elevated levels of C-reactive protein, total cholesterol, and triglycerides. Previous research has established a correlation between abnormal thyroid function and diminished cognitive abilities. The present study demonstrates that patients with CI exhibit reduced levels of free thyroxine and free triiodothyronine, as opposed to thyroid-stimulating hormone. Furthermore, an increase in D-dimer levels, the end product of cross-linked fibrin degradation, can serve as a sensitive indicator of heightened fibrinolysis and coagulation activity.^28^ This increased risk of thrombosis and coagulation in the brain can lead to white matter damage and subsequently impact cognitive function in patients. Finally, the findings from multiple logistic regression analysis indicated that age, education level, total cholesterol, and D-dimer were independent risk factors for CI among individuals with AF.

Left atrial diameter, left ventricular end-diastolic diameter, left ventricular end-systolic diameter, left atrial volume index, left ventricular ejection fraction, and peak early-diastolic velocity/peak late-diastolic mitral velocity ratio are all important parameters of cardiac function.^29^, ^30^, ^31^, ^32^ The correlation between the identification of cardiac function and alterations in brain structure and function resulting in CI has been established.^33^ Cardiac dysfunction leads to a decrease in ventricular blood output and sustained hypoperfusion of the brain, leading to neuron and glial cell damage and subsequent demise, ultimately affecting cognitive functions such as memory, cognition, executive ability, language expression, and emotional expression.^34^ Interestingly, a correlation analysis revealed a significant association between diminished cardiac function and impaired cognitive function. Qiu et al. demonstrated an elevated risk of approximately 80 % for Alzheimer's disease development in individuals with heart failure.^35^ Furthermore, a study conducted on heart failure patients indicated a decline in blood flow velocity within the middle cerebral artery among those with AF, resulting in overall cognitive decline and poor memory.^36^ Moreover, the presentinvestigation corroborated these findings by demonstrating that AF patients experiencing CI exhibited deficits in memory and attention. It is noteworthy that a substantial correlation exists between memory, attention, and parameters of cardiac function. This clinical research holds the potential to enhance clinicians' comprehension of the underlying pathophysiological mechanisms contributing to CI in individuals diagnosed with AF.

This study is constrained by several limitations. Firstly, the sample size is relatively small, which may impact the statistical power of the findings. Secondly, the study's duration only allows for the description of the causal relationship between cardiac function parameters and cognitive impairment in patients with atrial fibrillation, without further investigation into the long-term prognosis of these individuals. Additionally, the study exclusively focuses on patients with atrial fibrillation, limiting the generalizability of the results to other patient populations. Future research should focus on achieving comprehensive and multi-indicator joint grouping, incorporating additional CI evaluation techniques such as Mini-Cog, Menu Task, and Weekly Calendar Planning Activity. Designing a prospective multicenter observational cohort study is essential for predicting risk factor models for cognitive impairment in patients. Continuous monitoring of these factors can facilitate disease progression tracking and assess the relationship between cognitive decline and diminished cardiac function.

## Conclusion

The cardiac function parameters of patients are closely related to attention, orientation, memory, visuospatial and executive ability. Younger age, occupational situation, higher educational level, and lower levels of total cholesterol and D-dimer are protective against cognitive dysfunction. This study provides a clinical basis for clinical intervention studies in patients with AF combined with CI.

## Data available

Data is available from the corresponding author on request.

## Ethical approval

All procedures performed in this study involving human participants were in accordance with the ethical standards of the institutional and/or national research committee and with the 1964 Helsinki Declaration and its later amendments or comparable ethical standards. All subjects were approved by The First People's Hospital of Pinghu City (no. 201805ZJ5001).

## Consent to participate

Written informed consent was obtained from each subject.

## Consent for publication

Written informed consent for publication was obtained from all participants.

## Authors’ contributions

FengJiao Liao designed the research study. FengJiao Liao and ZongYi Hou performed the research. ZongYi Hou provided help and advice. ZongYi Hou analyzed the data. FengJiao Liao wrote the manuscript. FengJiao Liao and ZongYi reviewed and edited the manuscript. All authors contributed to editorial changes in the manuscript. All authors read and approved the final manuscript.

## Funding

Not applicable.

## Declaration of competing interest

The authors declare no conflicts of interest.
